# Single-Cell Microarray Chip with Inverse-Tapered Wells to Maintain High Ratio of Cell Trapping

**DOI:** 10.3390/mi14020492

**Published:** 2023-02-20

**Authors:** Ryota Sano, Kentaro Koyama, Narumi Fukuoka, Hidetaka Ueno, Shohei Yamamura, Takaaki Suzuki

**Affiliations:** 1 Division of Mechanical Science and Technology, Gunma University, Kiryu 376-8515, Japan; 2 Center for Advanced Medical Engineering Research & Development (CAMED), Kobe University, Kobe 650-0047, Japan; 3 Health and Medical Research Institute, National Institute of Advanced Industrial Science and Technology (AIST), 2217-14 Hayashi-cho, Takamatsu 761-0395, Japan

**Keywords:** single-cell, microarray, lab-on-a-chip, microfluidics Bio-MEMS, manipulation, high-throughput

## Abstract

A single-cell microarray (SCM) influenced by gravitational force is expected to be one of the simple methods in various fields such as DNA analysis and antibody production. After trapping the cells in the SCM chip, it is necessary to remove the liquid from the SCM to wash away the un-trapped cells on the chip and treat the reagents for analysis. The flow generated during this liquid exchange causes the trapped cells to drop out of conventional vertical wells. In this study, we propose an inverse-tapered well to keep trapped cells from escaping from the SCM. The wells with tapered side walls have a reduced force of flow toward the opening, which prevents trapped cells from escaping. The proposed SCM chip was fabricated using 3D photolithography and polydimethylsiloxane molding techniques. In the trapping experiment using HeLa cells, the cell residual rate increased more than two-fold for the SCM chip with the inverse-tapered well with a taper angle of 30° compared to that for the conventional vertical SCM chip after multiple rounds of liquid exchanges. The proposed well structure increases the number of trapped cells and decreases the cell dropout rate to improve the efficiency of cellular analysis.

## 1. Introduction

In conventional cellular analysis, cells are considered to be homogeneous, and the average characteristics from the analysis of a group of cells are used. However, various heterogeneities have been revealed, even in cells of the same species [1], and the analysis of single cells plays an important role in a wide range of biomedical applications, such as cancer diagnosis, DNA analysis, and the effects of drugs [2,3,4,5,6,7]. Therefore, various techniques have been developed to analyze single-cell behavior. Flow cytometry is the most commonly used method for single-cell analysis [8,9,10]. In this technique, cells are arranged in a line and passed in front of a laser beam through a sheath flow to be counted and sorted. Flow cytometry is widely used for high-throughput analysis because it allows the analysis of the physical and chemical properties of hundreds of thousands of cells per second. However, owing to the characteristics of the analytical method, it is impossible to follow specific cells and difficult to perform dynamic analysis. In contrast, fluorescence microscopy, which is a widely used analytical method, enables time-lapse measurements via the real-time observation and analysis of cells. In addition, it is possible to compare the cellular state before and after the analysis to compare the variation in response between cells. To observe a single cell with a microscope, a technique for arraying a large number of single cells is important [11,12,13,14,15]. For example, single-cell analysis methods using microscope-based microarrays have been proposed for high-throughput single-cell analysis in immunology and other fields [16].

As mentioned above, for microscopic analysis, it is necessary to isolate single cells from a large population of cells and trap them at a specific location. Techniques for trapping single cells generally include optical, electrical, acoustic, hydrodynamic, and gravitational force methods. An optical tweezer is formed by focusing a laser beam which generates an electric field with a strong gradient to attract charged cells and trap them at the designated position [17]. Multiple cells can be simultaneously manipulated using multiple lenses and mirrors. Although optical tweezers are non-contact, high-resolution cell manipulators, damage (caused by the high-power laser) to cells and biomolecular activity remains a practical issue for bioanalysis. Dielectrophoresis (DEP) utilizes the dielectric properties of cells to trap them. Electrodes are placed on one or both sides of a flow channel; cells are passed through the nonuniform electric field, which manipulates the charged cells and separates and traps them at the designated position (s) [18,19,20]. DEP also has high-resolution cell manipulation performance; however, specialized knowledge and complex equipment are required for a well-balanced set up, considering the fluid, electrical, and gravitational forces. The acoustic method traps cells in a channel using acoustic radiation force [21,22]. This method is less invasive than light or electrical methods but is less manipulatable because of the low diffusivity and sphericity of cells. The hydrodynamic method applies microfluidic device technology to trap cells in a designated position, using a well in a channel or a specific channel shape, by flowing a cell suspension through a microchannel (microfluidic channel) [23,24,25]. This method is also minimally invasive; however, the number of traps is limited due to the need for complex channel structures, pumps, and valves. The trapping method using gravitational force is called Single-Cell Microarray (SCM) [16,26]. As SCM is a passive method in which cells are trapped using gravitational force by seeding the cell suspension, it has been applied in a variety of ways as a minimally invasive and simple trapping method [27,28].

In passive SCM, the important factors to consider when determining the well geometry are the well dimensions and shape. The two-dimensional geometry of a conventional well fabricated using photolithography is flexible in design [29,30,31]. In contrast, we propose an SCM with inverse-tapered three-dimensional (3D) wells (fabricated via 3D photolithography) to achieve single-cell immobilization and prevent cell dropout during the liquid exchange [32]. We carried out immobilization experiments with protoplasts of single plant cells with a mean diameter of 30 μm and found that the inverse-tapered wells contributed to a higher cell residual rate after one wash than the vertical wells.

In this study, we propose an SCM with inverse-tapered 3D wells for human cells to retain trapped single cells during multiple rounds of liquid exchanges. The proposed SCM chip was fabricated using 3D photolithography, and the effects of the opening diameter and tapered angle of the wells on cell dropout were evaluated. The proposed SCM chip is operated simply by introducing the cell suspension; the 3D well geometry continuously traps single cells via gravitational force.

## 2. Materials and Methods

### 2.1. Principle of Single-Cell Microarray Chip

In this study, the cells were trapped in wells via gravity settling. When cell suspensions are seeded on the SCM and left to stand for a certain period of time, the cells settle because they are denser than the liquid. Therefore, without any special manipulation, the cell suspension was seeded, and the cells settled. Those with a center of gravity above the well opening entered the well and were trapped.

**Figure 1:**
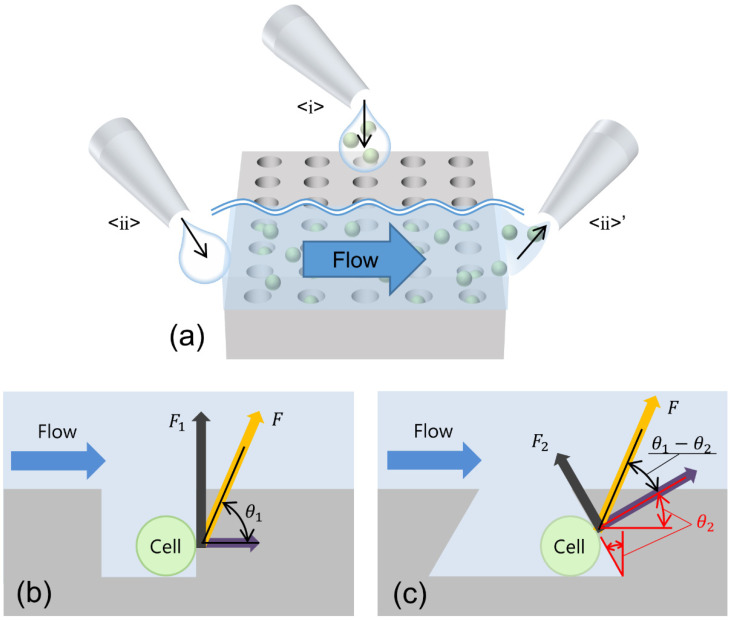
Schematics of a well of the SCM driven by gravitational force. (**a**) Operations of the SCM chip: <ⅰ> trapping cells by seeding the cell suspension; <ⅱ>–<ⅱ>’ flow generated by liquid exchange. (**b**) Vertical well. (**c**) Inverse-tapered well. Both schematics represent the component force when a drag force of the same magnitude *F* is applied to the cell at the same angle to the bottom.

After trapping, it was necessary to wash away the cells that were not trapped in the wells and were left on the top surface of the SCM to allow the introduction of reagents for cell response measurements. During these operations, a flow was generated in the wells; it pushed up the cells. The push-up force can be divided into two vectors: one perpendicular to the sidewall and another toward the opening. In a typical vertical well, as shown in Figure 1b, the cells are pushed up and dropped out of the well by the vector $F_{1}$ in the direction of the opening. If the wall of the well is inclined, as shown in Figure 1c, the opening direction vector $F_{2}$ becomes as follows:

(1) $$F_{2}=F_{1}\sin\left(\theta_{1}-\theta_{2}\right),$$

where $\theta_{1}\left(>0\right)$ is the drag force due to the flow field and is assumed to be constant. If the angle of the tapered wall $\theta_{2}\left(>0\right)$ is increased, $\sin\left(\theta_{1}-\theta_{2}\right)\left(>0\right)$ becomes smaller. As $F_{2}$ in the opening direction becomes smaller with a larger tapered angle, cell dropout is suppressed.

In passive SCM, the important factors to consider when determining the well geometry are the well dimensions and shape. In this study, human cervical cancer cells (HeLa) were used for the chip design. The diameter of the HeLa cells ranged from 11 to 13 µm. To determine the optimal shape of the wells to trap a single cell, we prepared three types of wells with opening diameters of 10, 15, and 20 μm. If the opening diameter is equal to the cell diameter, then only one cell can enter and be trapped in the well. A well with a larger opening diameter achieved a higher probability of trapping cells and more cells. However, if the opening diameter is too large, the probability of trapping multiple cells in the well increases. Hence, the opening diameter was designed to be at a size between 1 and 2 times the cell diameter.

**Figure 2:**
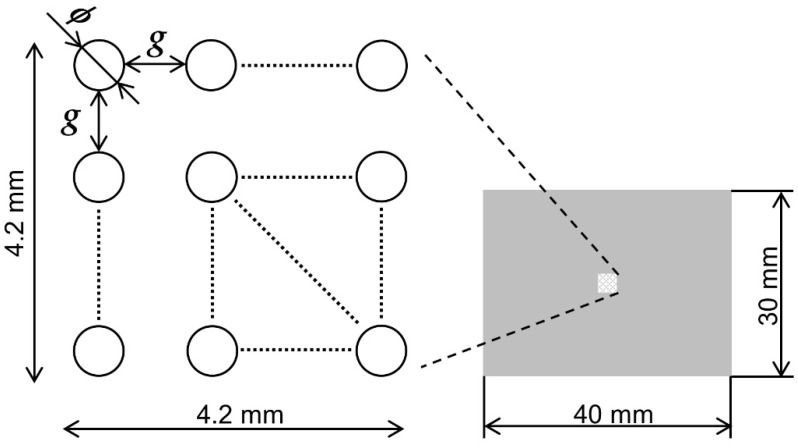
Design of the SCM chip. A trapping area of 4.2 mm × 4.2 mm is centered on a chip of 30 mm × 40 mm. There are 9 types of wells designed as trapping structures with combinations of dimensions between the opening diameter *ϕ* of 10, 15, and 20 μm and gap *g* of 25, 30, and 35 μm.

Shortening the gaps between the wells and placing high concentrations of wells in the trapping area rendered single cells easier to trap. In addition, the amount of sample required was reduced. Therefore, to evaluate the effect of the gaps between wells on cell trapping, we set up three types of gaps between wells at intervals of 5, 25, 30, and 35 μm. The wells used in this study had tapered walls. Because the bottom of the well is larger than the top opening of the well, the diameter of the bottom is determined using Equation (2):

(2) $$d_{b}=d+2h\tan\theta_{r},$$

where *d* is the opening diameter, $d_{b}$ is the diameter of the bottom, *h* is the depth of the well, and $\theta_{r}$ is the tapered angle of the sidewall. The designed prototype had a well depth of 15 μm and a maximum sidewall tapered angle of 30°, which increased the bottom diameter by up to 17.2 μm relative to the opening diameter. The minimum distance between wells was set to 25 μm because the distance between wells must be greater than the increase in the bottom diameter when fabricating complex 3D shapes. Based on the above, nine SCM chip geometries were designed, as shown in Figure 2.

### 2.2. Fabrication of SCM Chip

**Figure 3:**
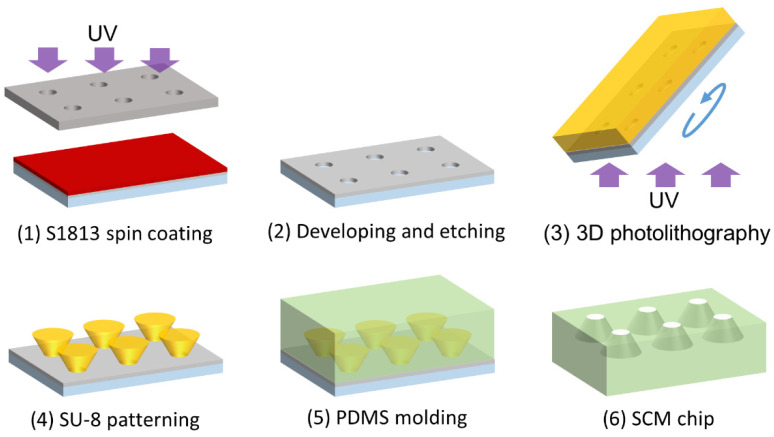
Fabrication procedure of the SCM chip using 3D photolithography.

A micromold composed of thick negative photoresist (SU-8, Nippon Kayaku Co., Ltd., Tokyo, Japan) was fabricated on a glass substrate using 3D photolithography and molded using polydimethylsiloxane (PDMS; SILPOT 184 W/C, Dow Toray Co., Ltd., Tokyo, Japan) to fabricate the SCM chip. Figure 3 presents an overview of the fabrication process.

First, the mask pattern was transferred onto a glass substrate for backside exposure. Because the mask pattern includes a fine pattern, accuracy cannot be obtained with the normal contact exposure. Therefore, considering the adhesion between the substrate and the mold, backside exposure was used. A Cr film with a thickness of 200 nm was deposited onto the glass substrate, which had been cleaned with sulfuric acid-permeated water, using a sputtering system at a power of 200 W for 5 min. A positive photoresist (MICROPOSIT S1813, Rohm and Haas Electronic Materials K.K., Niigata, Japan) was coated onto the Cr-patterned substrate using a spin coater (1H-DX2, Mikasa Corp., Hiroshima, Japan). Next, exposure was performed with an exposure machine (Ushio Inc., Tokyo, Japan) using a photomask with the mask pattern depicted in Figure 2. The pattern was developed using a photoresist developer (NMD-3, Tokyo Ohka Kogyo Co., Ltd., Kawasaki, Japan), and Cr was etched using a Cr-etching solution (Chrome Etching TK, Hayashi Pure Chemical Ind., Ltd., Taki-cho, Japan).

Next, an adhesion layer composed of SU-8 (SU-8 3005, Nippon Kayaku Co., Ltd.) diluted with thinner (SU-8 Thinner, Nippon Kayaku Co., Ltd.) was formed on the Cr-patterned glass substrate. After spin coating, the resist was heated on a hot plate to dry completely. The entire surface of SU-8 on the glass substrate was exposed to 400 mJ/cm^2^ and heated on a hot plate to completely cure the film, resulting in an adhesion layer of approximately 1 μm thickness. The adhesion layer prevented the SU-8 microstructures fabricated on the adhesion layer from peeling off together with PDMS during demolding.

**Figure 4:**
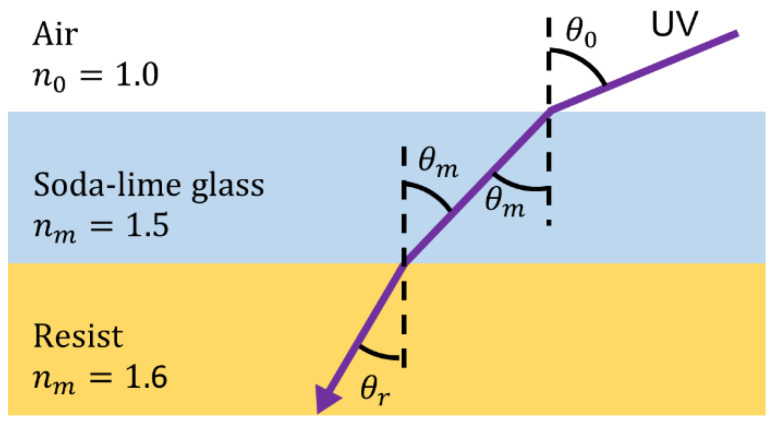
Relationship between incidence angle and tapered angle.

Next, inverse-tapered 3D wells composed of SU-8 were fabricated using 3D photolithography on the adhesion layer [33]. After the surface treatment of the cured adhesion layer via reactive ion etching (RIE; ES401, Nippon Scientific Co., Ltd., Tokyo, Japan), multiple layers of SU-8 3005 were applied to achieve a thickness of 15 μm with respect to the thickness of one cell. The first layer was spun at 1000 rpm using spin-coating to deposit a film thickness of 10 μm before baking, and the second layer was spun at 800 rpm to deposit a film thickness of 5 μm before baking, resulting in a film with an overall thickness of 15 μm. Subsequently, 3D photolithography was used to expose the Cr-patterned glass substrate from the back side at an exposure dose of 400 mJ/cm^2^. Furthermore, 3D photolithography was performed by fixing the substrate coated with a resist on an inclined table and rotating it while exposing it from the back side of the glass substrate. At this time, as shown in Figure 4, if the incident angle of light $\theta_{0}$ is the same as the inclined angle of the stage, the refractive index of air is $n_{0}$, the refractive index of glass is $n_{m}$, and the refractive index of the resist is $n_{r}$, the tapered angle $\theta_{r}$ of the fabricated structure is determined by Snell’s law as follows:

(3) $$\theta_{r}=\sin^{-1}\left(\frac{n_{0}}{n_{r}}\sin\theta_{0}\right).$$

The refractive index of SU-8 was set to $n_{r}=1.6$. To fabricate structures with vertical side walls and tapered angles of 10, 20, and 30°, the inclined angle of the exposure stage calculated from Equation (3) was fixed at 0, 16.1, 33.2, and 53.2°, respectively. After a post-exposure bake (PEB), the microstructures were developed using an SU-8 Developer. The SU-8 micromold was fabricated using the above process.

Finally, PDMS SCM chips were fabricated using replica molding. A release agent (Barrier Coat, Shin-Etsu Chemical Co., Ltd., Tokyo, Japan) was spin-coated onto the fabricated SU-8 micromold. Liquid PDMS (with main agent:hardener = 10:1) was poured on top of the mold and vacuum-degassed. After degassing, the PDMS was heated at 80 °C for 2 h to cure and then was slowly peeled off from the mold to complete the PDMS SCM chip.

### 2.3. Cell Culture and Concentration Tuning of Cell Suspension

As shown in Figure S1, HeLa cells derived from human cervical cancer cells were used in the cell trapping experiment [34,35,36]. For fluorescence observation using a microscope (IX71, Olympus), the presence of cells was determined based on the fluorescence emitted by the cells. The HeLa-H2B cell line was cultured in Dulbecco’s modified Eagles medium (DMEM) supplemented with a 10% fetal bovine serum (FBS) and 1 mL of penicillin streptomycin. The cells were grown in 5% carbon dioxide (CO_s_) in a humidified incubator at 37 °C until 70–80% confluence. 

The concentration of the cell suspension seeded onto the SCM chip was an important operating parameter for determining the cell trapping rate. Because the number of wells per chip varied according to the opening diameter and the gap between wells, the cell concentration was adjusted to provide a constant theoretical probability of cell trapping in each geometry.

Because cells sink downward due to gravity, the probability *p* of a cell becoming trapped in one well is expressed by the ratio of the opening hole area *Ao* to the entire top area *A* of the chip as follows:

(4) $$p=\frac{A_{o}}{A}.$$

If the number of wells trapping a cell on the chip is *N_t_*, the number of cells seeded on the chip is *n_s_*, and all seeded cells are trapped sequentially in the wells with probability *p*, the following equation is obtained:

(5) $$N_{t}=n_{s}p.$$

Substituting Equation (4) into Equation (5), the number of cells *n_s_* that need to be seeded on the chip is as follows:

(6) $$n_{s}=N_{t}\frac{A}{A_{o}}.$$

Next, the theoretical cell-trapping rate in the above condition and the chip geometry is calculated. If the probability *f*(*m*) as a function of the number of cells above one well on chip *m* is represented by a binomial distribution, then the average number *μ* of cells above one well is given by the following equation from the property of a binomial distribution:

(7) $$\mu =n_{i}\frac{1}{N},$$

where *N* is the number of wells on the chip, and *n_i_* is the total number of cells above all wells. Here, *n_i_* is very large, whereas *μ* is a very small number; therefore, the binomial distribution is rewritten as a Poisson distribution, and the probability *f*(*m*) is expressed as

(8) $$f\left(m\right)=\frac{\mu^{m}\times e^{-\mu }}{m!}.$$

Assuming that *μ* = 1 for all seeded cells are individually trapped to enter each well, the probability of single-cell trapping for all wells in the SCM chip is obtained by subtracting the probability of *m* = 0 for no cells from the total trapping rate (100%). This is expressed as follows:

(9) $$1-f\left(0\right)=1-\frac{1^{0}\times e^{-1}}{0!}=0.63.$$

Because a single cell was trapped in 63% of the wells, the required cell concentration *C* of the cell suspension was obtained using the following equation:

(10) $$C=\frac{n_{s}}{V}=N_{t}\frac{A}{A_{o}}\cdot \frac{1}{V}=0.63N\frac{A}{A_{o}}\cdot \frac{1}{V},$$

where *V* is the drop volume of the suspension. To increase the trapping rate, the average number *μ* of cells above the wells should be increased. In other words, the total number of cells seeded on the chip should be increased. However, because the volume in the wells is increased by the tapered structure, which increases the trapping of multiple cells, *μ* = 1 was assumed in this study.

### 2.4. Cell Trapping Test

The surface of the fabricated SCM chip was hydrophilized with a reactive ion etching (RIE) machine (FA-1, Samco). Because the single-cell occupancy increased when the contact angle decreased [16], the surface of the entire chip was treated by RIE exposure. Then, a 2 mm thick silicone sheet with a 6 mm diameter hole drilled with a biopsy trepan (BP-60F, AS ONE Corp., Osaka, Japan) was attached to form a wall surrounding the trapping area. The confluent HeLa-H2B cells were detached from the petri dish with trypsin, and the cell suspension of 100 μL was dropped onto the SCM chip, with the cell concentration adjusted for each opening diameter, as shown in Table 1. The cells were trapped in the wells via gravitational settling for 5 min. Because the area *A* of trapping is constant, as shown in Equation (6), only the opening diameter affects the ease of cell trapping. Therefore, the concentration of the cell suspension varied for each opening diameter condition, as shown in Table 1. Cells that remained on the SCM without trapping were washed away several times with phosphate buffer solution (PBS). The number of single cells trapped in the SCM were counted at the center and four corners of the array using a fluorescence microscope and image analysis. The average value was used as the result of the array. Three arrays were tested for each term. 

The single-cell occupancy in Equation (11) was used to evaluate the initial trapping efficiency of cells after seeding. SCM chips with different opening diameters, gaps between wells, and arrays with different tapered angles were tested, and the single-cell occupancy rates were calculated as follows:

(11) $$\mathrm{Single}\;\mathrm{cell}\;\mathrm{occupancy}\left[\%\right]=\frac{\mathrm{Number}\;\mathrm{of}\;\mathrm{wells}\;\mathrm{with}\;\mathrm{only}\;\mathrm{one}\;\mathrm{cell}\;\mathrm{trapped}}{\mathrm{Number}\;\mathrm{of}\;\mathrm{wells}\;\mathrm{in}\;\mathrm{an}\;\mathrm{image}}\times 100.$$

Because the influence of the opening diameter is the probability of cells existing above the opening, we assumed that the influence is similar in vertical and inverse-tapered wells. Hence, we investigated the influence of opening diameter only in the vertical wells.

To evaluate the maintenance of trapping cells in inverse-tapered wells, a liquid exchange experiment was performed using the SCM chip with the optimal opening diameter evaluated in the vertical wells. We carried out the experiment three times for each tapered angle. As in the initial trapping experiment, multiple washing steps with PBS were performed after the cell trapping. Although the flow rate is to affect the cell dropout from the well, the SCM using gravitational force is characterized by a simple operation using a pipet. Therefore, the flow rate was not controlled, and the inflow of the suspension was constant based on the pipette operation assuming actual use. Images at odd times of PBS washing were taken with a fluorescence microscope, and the cell residual rate in Equation (12) was calculated from the images as:

(12) $$\begin{array}{l} \mathrm{Cell}\;\mathrm{residual}\;\mathrm{rate}\left[\%\right]\\ \;=\frac{\mathrm{Number}\;\mathrm{of}\;\mathrm{wells}\;\mathrm{with}\;\mathrm{one}\;\mathrm{cell}\;\mathrm{trapped}\;\mathrm{after}\;\mathrm{liquid}\;\mathrm{exchange}}{\mathrm{Number}\;\mathrm{of}\;\mathrm{wells}\;\mathrm{with}\;\mathrm{one}\;\mathrm{cell}\;\mathrm{trapped}\;\mathrm{before}\;\mathrm{liquid}\;\mathrm{exchange}}\times 100. \end{array}$$

## 3. Results and Discussion

### 3.1. Characterization of SCM Chip

**Figure 5:**
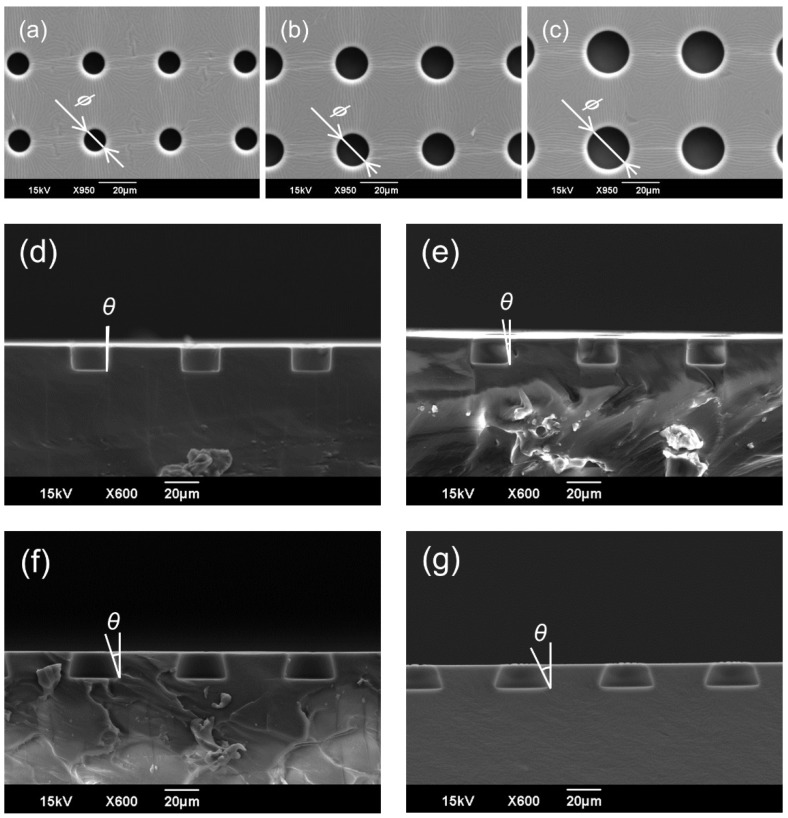
SEM images of the fabricated SCM chips. (**a**) Top view of a vertical well with an opening diameter of 10 μm and a gap of 30 μm. (**b**) Top view of a vertical well with an opening diameter of 15 μm and a gap of 30 μm. (**c**) Top view of a vertical well with an opening diameter of 20 μm and a gap of 30 μm. (**d**) Cross-sectional view of a vertical well with an opening diameter of 20 μm and a gap of 30 μm. (**e**) Cross-sectional view of an inverse-tapered well with an opening diameter of 20 μm, a gap of 30 μm, and a tapered angle of 10°. (**f**) Cross-sectional view of an inverse-tapered well with an opening diameter of 20 μm, a gap of 30 μm, and a tapered angle of 20°. (**g**) Cross-sectional view of an inverse-tapered well with an opening diameter of 20 μm, a gap of 30 μm, and a tapered angle of 30°.

Figure 5 shows the results of the Scanning Electron Microscopy (SEM; JCM-5700LV: JEOL) observations of the SCM chip fabricated via PDMS molding. The released PDMS SCM chip was cut, and the opening diameter and tapered angle were measured from the cross-sectional images of the wells. As shown in Table 2 and Table 3, the opening diameter and tapered angle were measured for three randomly selected wells on the chip. The errors of the opening diameter and tapered angle were approximately 5 and 10% for all chip geometries, respectively. Table 2 shows that the opening can be fabricated with high accuracy for all the sizes. During fabrication, a mask pattern was transferred onto a glass substrate, and a resist film, which serves as the micro-mold structure, was directly deposited on top of the mask pattern. Because the opening was on the glass substrate side of the deposited resist, the mask and resist had good contact during exposure, resulting in high-precision fabrication. Table 3 shows that the discrepancy between the design values and fabrication results for the tapered angle increased with the design value. As the angle increased, some of the light was reflected more on the glass substrate, and as a result, the exposure amount was reduced, and the exposure at the corners of the well bottom tended to be insufficient. In addition, the larger the tapered angle, the more difficult it was for the developer solution to penetrate the gaps between the structures; therefore, angle errors occurred more often due to the lack of development. Because the combination of 3D photolithography and PDMS molding were used to fabricate the SCM chips for cell trapping tests with various tapered angles, the errors for all tapered angles were less than 10%. When a replicated PDMS was peeled off from the SU-8 micromold, it was predicted that the inverse-taper shape would render it difficult to remove the mold and that the mold would break. In this study, the mold release property was improved by applying a mold release agent, and the mold strength was increased by the adhesion layer so that the mold was released with the original flexibility of PDMS. The fabricated micromolds were used repeatedly in conventional soft lithography.

### 3.2. Cell Trapping Experiments

**Figure 6:**
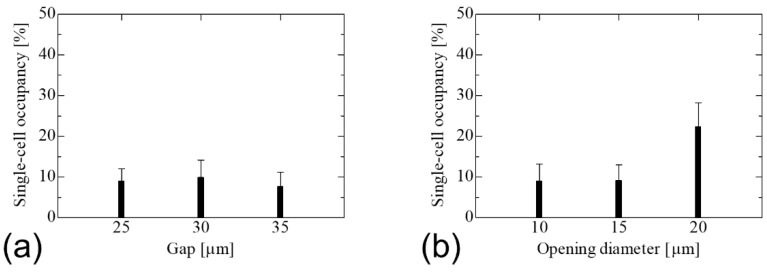
Single-cell occupancy of the SCM chip with vertical wells. (**a**) In the gap change between wells with an opening diameter of 10 μm, the single-cell occupancy shows no change with the gap. (**b**) In the opening diameter change with a gap of 25 μm, single-cell occupancy increased with increasing opening diameter, reaching a maximum at 20 μm of the opening diameter.

Figure 6a shows the results of cell trapping using SCM chips with an opening diameter of 10 μm and gaps of 25, 30, and 35 μm. The single-cell occupancy remained almost the same under all conditions. As the probability of trapping for each gap did not change in the calculations of cell suspensions, the gap between wells had no effect on the ease of cell trapping. Figure 6b shows the results of the cell trapping experiments using SCM chips with a gap of 25 μm and opening diameters of 10, 15, and 20 μm. The chip with an opening diameter of 20 μm exhibited the highest single-cell occupancy. An opening diameter that is sufficiently larger than the cell diameter is effective for efficient cell trapping. The opening diameter of 20 μm was the largest among the SCM chips used in this study and therefore had the highest probability of trapping cells, while the HeLa-H2B cells of 10 to 14 μm in size were the most efficiently trapped single cells because they were not large enough to trap multiple cells. In addition, when cells are trapped in the wells, they must push away the liquid previously filled in the wells by their own weight. Because the self-weight, which is the force required for cell entry into the well, is constant regardless of the well shape, it is believed that the larger the opening diameter relative to the cells, the larger the gap during entry and the easier it is for the pushed-out liquid to be expelled, resulting in easier cell entry into the well.

**Figure 7:**
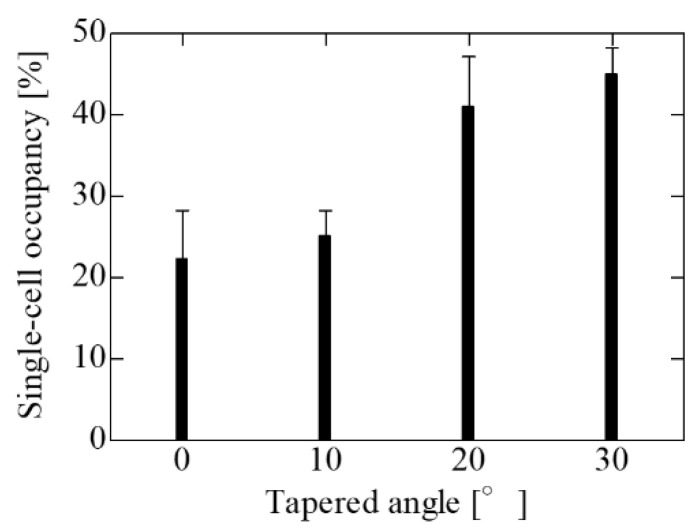
Single-cell occupancy in the change in the tapered angle on the SCM having the opening diameter of 20 μm and the gap of 30 μm. Single cell occupancy increased with increasing tapered angle, reaching a maximum at an angle of 30°. Single-cell occupancy in the change in the tapered angle on the SCM having an opening diameter of 20 μm and a gap of 30 μm. A single-cell occupancy increased with increasing tapered angle, reaching a maximum at an angle of 30°.

Figure 7 shows the results of the trapping experiments using SCM chips with different tapered angles and an opening diameter of 20 μm, the optimal opening diameter evaluated in the vertical wells. The single-cell occupancy increased with the tapered angle, and the largest single-cell occupancy was observed for the SCM chip with the largest taper angle of 30°. Because the opening diameter was the same regardless of the angle, it did not change the initial trapping probability. However, cell dropout due to the flow during PBS washing before imaging was suppressed on the SCM chip with a larger taper angle. Then, the cells remaining un-trapped on the SCM chip were trapped by the liquid flow during PBS washing. Therefore, the single-cell occupancy of the SCM chip with inverse-tapered wells is improved compared to that of the SCM chip with vertical wells. The results show that a further increase in the tapered angle can be expected to improve the single-cell occupancy. However, because 3D photolithography is based on Snell’s law, the maximum tapered angle of the well wall is 38° based on total reflection. If the tapered angle is too large, there are disadvantages such as difficult edge processing, an increase in the dead volume of the wells, and a decrease in the number of holes inside the trapping area due to the long bottom edge. Therefore, a tapered angle of 30° is the optimum angle due to the fabrication yield and the fabrication limit of the tapered angle. Although the concentration of cell suspension was adjusted to achieve a theoretical single-cell occupancy of 63%, the maximum single-cell occupancy in the experiment was approximately 45%. During the experiment, a silicon sheet wall was placed on the SCM chip to maintain suspension in the trapping area. When the suspension was dropped, the cells gathered at the edge of the wall due to surface tension, and the number of cells in the trapping area was smaller than the theoretical number on the SCM chip, which may have reduced the single-cell occupancy. In addition, because the actual cell size varied, the single-cell occupancy was smaller than the theoretical occupancy due to the presence of wells trapping multiple cells or un-trapped cells larger than the wells.

**Figure 8:**
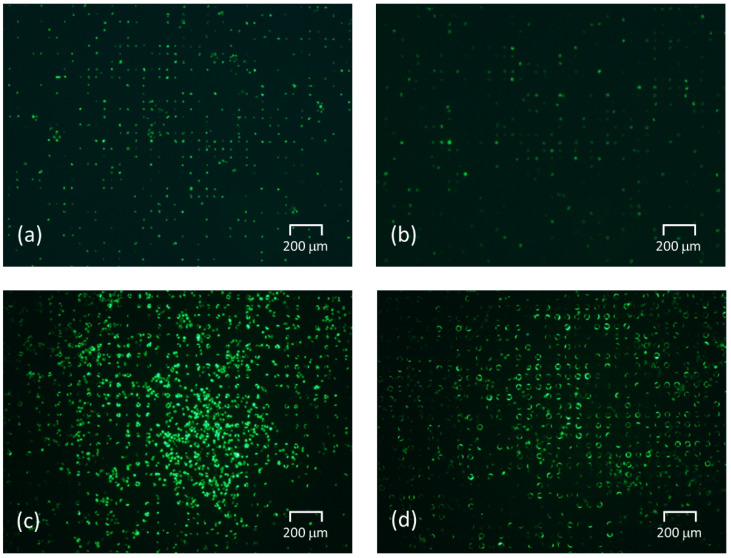
Fluorescent images of trapped cells using the fabricated SCM chip (ISO 200/exposure time 5 s). (**a**) Before liquid exchange experiment using the vertical wells. (**b**) After 11 liquid exchanges using the vertical wells. (**c**) Before the liquid exchange experiment using the tapered wells inclined at 30°. (**d**) After 11 liquid exchanges using the tapered wells inclined at 30°.

**Figure 9:**
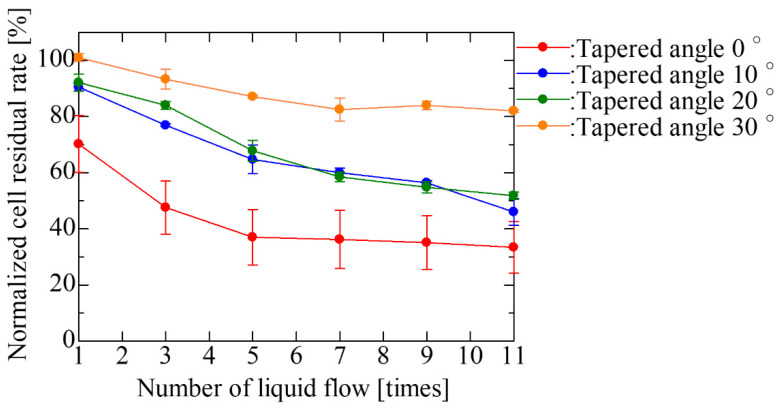
Normalized cell residual rate with repeated liquid exchange of the SCM chip having an opening diameter of 20 μm and a gap of 30 μm. The cell residual rate normalized with respect to the result at a tapered angle of 30° at one liquid exchange is plotted for an odd number of liquid exchanges.

Liquid exchange was performed for each tapered angle in the SCM chip with an opening diameter of 20 μm and a gap of 30 μm, which was the optimal shape based on the results of single-cell occupancy. Figure 8 and Figure 9 show the fluorescent images of trapped cells using the fabricated SCM chip and the normalized cell residual rate using repeated liquid exchange for the SCM chip. We carried out 11 times liquid exchanges as the number of exchanges required to achieve convergence of the cell residual rate in the vertical well. As described above, the larger the tapered angle, the more cells were trapped at the beginning, and the fewer cells dropped out even after repeated PBS washes, and the cells continued to be trapped. After multiple liquid exchanges, the number of final residual cells was more than twice as high. In the trapping experiment, the larger the tapered angle became, the greater the difference in single-cell occupancy after liquid exchange, due to the decrease in the number of cells dropped by the flow and the re-trapping of free cells remaining on the chip. Thereafter, repeated washing almost eliminated cells remaining un-trapped in the chip, and the larger the tapered angle, the more the dropout was suppressed, and the smaller the decrease rate of the cells. In addition, the larger the tapered angle, the less the influence of the flow during washing; thus, the error caused by the manipulation was reduced, and the cell trapping was found to be highly reproducible. However, it was observed that not only cells but also liquid was difficult to remove and that a large amount of liquid remained in the wells after aspiration. This may inhibit cells from entering the wells. In the near future, it is expected that the wells will be designed with a structure that allows only liquid to drain out.

## 4. Conclusions

In this study, we proposed an SCM chip for human-derived cells, which efficiently traps single cells and maintains trapped cells against liquid turbulence in the chip. We designed a well with an inverse-tapered structure with inclined side walls and conducted cell trapping experiments with HeLa cells using SCM chips fabricated by 3D photolithography and PDMS molding.

In the cell trapping experiment using SCM chips with varying gaps between vertical wells, the single-cell occupancy was not changed. In the experiments using SCM chips with vertical wells of varying opening diameters, single-cell occupancy increased with the opening diameter, reaching a maximum at a diameter of 20 μm in the tested conditions. In the SCM chip with inverse-tapered wells, single-cell occupancy increased with the taper angle. The cell residual rate also increased with taper angle in multiple liquid exchange experiments. After multiple rounds of liquid exchanges, the cell residual rate of the SCM chip with the inverse tapered wells at a 30° angle was more than two times higher than that of the conventional SCM chip with vertical wells. Hence, the proposed SCM chip with inverse-tapered wells is effective for efficient cell trapping and the maintenance of cell trapping. 

## Figures and Tables

**Table 1:** Concentrations of cell suspension relative to geometry of SCM chip.

Opening Diameter [μm]	Concentration of Cell Suspension [/μL]
10	2.25 × 10^3^
15	1.00 × 10^3^
20	0.56 × 10^3^

**Table 2:** Opening diameters of vertical wells with a gap between wells of 30 μm.

Design Value *ϕ* [μm]	Measured Value *ϕ* [μm]	SD [μm]
10	10.4	0.4
15	15.7	0.4
20	20.7	0.6

**Table 3:** Tapered angles of the inverse-tapered wells with an opening diameter of 20 μm and a gap between wells of 30 μm.

Design Value *θ* [°]	Measured Value *θ* [°]	SD [°]
0	1.0	0.2
10	8.8	0.3
20	18	0.6
30	27	1.6

## Data Availability

The data supporting these study findings are available from the corresponding authors on reasonable requests.

## Supplementary Materials

The following supporting information can be downloaded at: https://www.mdpi.com/article/10.3390/mi14020492/s1, Figure S1: Images of HeLa-H2B cells used for cell-trapping experiments.
