# Supplementary material for: Single-Cell Microarray Chip with Inverse-Tapered Wells to Maintain High Ratio of Cell Trapping
Source: Micromachines (Basel). 2023 Feb 20;14(2):492. doi: 10.3390/mi14020492 (PMC9959924; doi:10.3390/mi14020492)
Supplement: Supplementary file 1 [file micromachines-14-00492-s001.zip › micromachines-2197901-supplementary.pdf]

micromachines

Supplementary Materials:

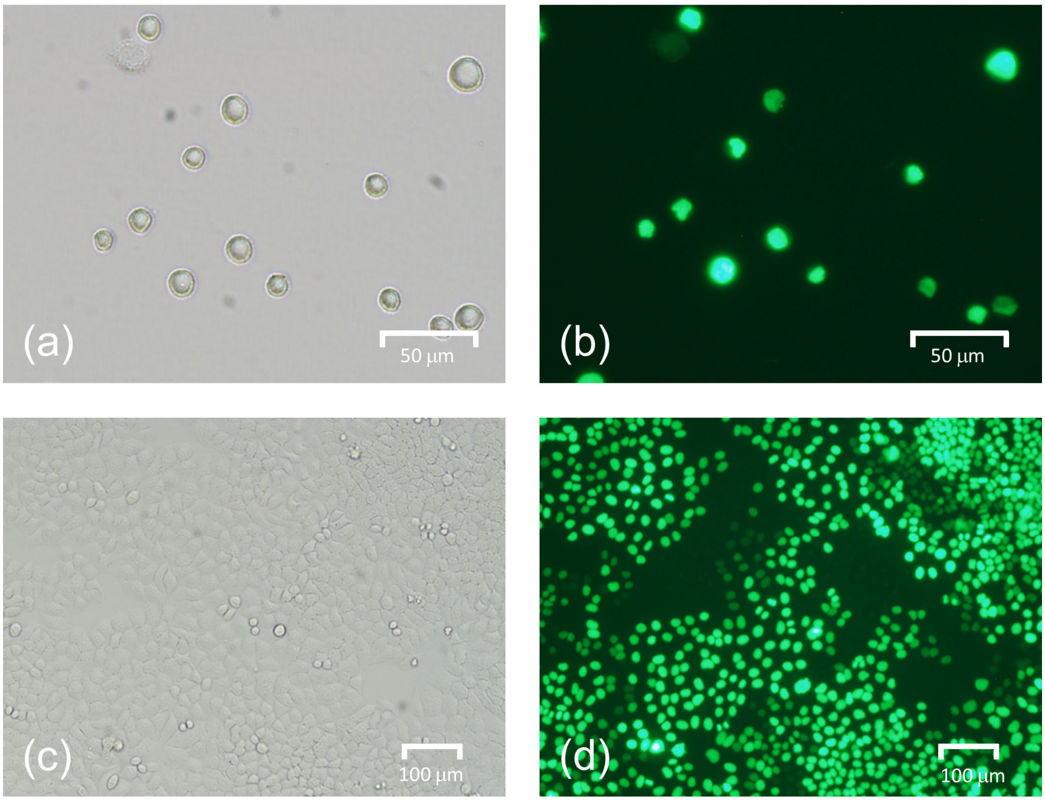

Figure S1. Images of HeLa-H2B cells used for cell-trapping experiments. The nuclei of HeLa cells were stained green fluorescence. These cells had been treated with histones, which were components of eukaryotic chromosomes with cell nuclei [34,35]. Histones were composed of four types, one of which, H2B, was fused with green fluorescent protein (GFP) to enable fluorescence observation [36]. (a) Bright-field image of HeLa-H2B cells before culture. (b) Fluorescent image of HeLa-H2B cells before culture. (c) Bright-field image of confluent HeLa-H2B cells after culture. (d) Fluorescent image of confluent HeLa-H2B cells after culture.
